# CASE REPORT Use of a Hydroconductive Dressing to Treat a Traumatic Avulsive Injury of the Face

**Published:** 2012-07-25

**Authors:** Colin Jerome Perumal, Martin Robson

**Affiliations:** ^a^Department of Maxillofacial and Oral Surgery, Medical University of South Africa, University of Limpopo, Ga-Rankuwa, Gauteng, Republic of South Africa; ^b^University of South Florida, Tampa, Florida

## Abstract

**Introduction and objective:** Traumatic avulsive injuries present complex therapeutic decisions. Radical and repeated debridement of all foreign material, necrotic tissue, bacteria, and deleterious chemicals followed by control of the bacterial bioburden and wound closure has been the gold standard. However, when such injuries occur in the face, the treatment requires modification. Specialized structures, nerves, and a maximum amount of tissue must be preserved. Topical antimicrobials may lead to dessication and further injury to tissue. Therefore, alternative treatments must be considered. Recently, a hydroconductive dressing has been demonstrated to decrease edema by removing excess exudate, to remove debris and necrotic tissue, and to decrease bacteria and deleterious cytokines in wounds. **Methods:** Regular dressings were done between 1 and 3 days by dedicated personnel, using a hydroconductive dressing. Following an initial conservative debridement and reconstruction while attempting to preserve as much of the normal structure as possible, the wounds were dressed with a hydroconductive dressing. **Results:** Using only selective conservative debridement following bony reconstruction and repeated hydroconductive dressing changes, this severe injury healed with preservation of the important facial features. No further extensive surgical procedures were required. On discharge, the patient was able to function well with a reasonably good aesthetic result. She was subsequently lost to follow-up. **Conclusion:** This case report demonstrates that a hydroconductive dressing can be useful for traumatic avulsive injuries.

Deterrents to wound healing in traumatic injury cases include inflammation, edema, decreased blood supply, necrotic tissue, and a significant bioburden.^1^ This bioburden is detrimental to healing when tissue levels of bacteria exceed 10^5^ CFUs per gram of tissue or contain β-hemolytic streptococci.^1^ In addition to bacteria that have attained a tissue level, microbes residing on the surface of a wound can organize as a biofilm community.^2^ Inflammation and edema are necessary to provide a source of nutrition for a biofilm community. Also, wound bacteria kill and digest host tissue for additional nutrition. Bacteria and fungi utilize inflammation to produce plasma exudates that percolate through the biofilm, thus providing sustainable nutrition for the entire community.^3^

Because of the deterrents discussed, traumatic avulsive injuries present complex therapeutic decisions. Radical and repeated debridement of all foreign material, necrotic tissue, bacteria, and deleterious chemicals followed by control of the bacterial bioburden and wound closure has been the gold standard. However, when such injuries occur in the face, the treatment requires modification. Specialized structures, nerves, and a maximum amount of tissue must be preserved. Topical antimicrobials may lead to dessication and further injury to tissue. Therefore, alternative treatments must be considered. Recently, a hydroconductive dressing has been demonstrated to decrease edema by removing excess exudate, to remove debris and necrotic tissue, and to decrease bacteria and deleterious cytokines in wounds.^4^ If rapid removal of inflammatory exudates aids in diminishing wound biofilms' pathogenic activity, the wound healing trajectory may be returned toward normal.

A case report is presented to show the effectiveness of this hydroconductive dressing in the treatment of a severe traumatic avulsive injury to the face.

## METHODS

A 39-year-old woman presented to the emergency department 8 days after being involved in a motor vehicle accident in which a metal bar protruding out of another vehicle had rammed into the left side of her face. There was severe disruption of soft tissue architecture of the left face with a malodorous septic wound consisting of loose bone fragments, grass, pieces of glass, stone, and sand.

**Figure 1:**
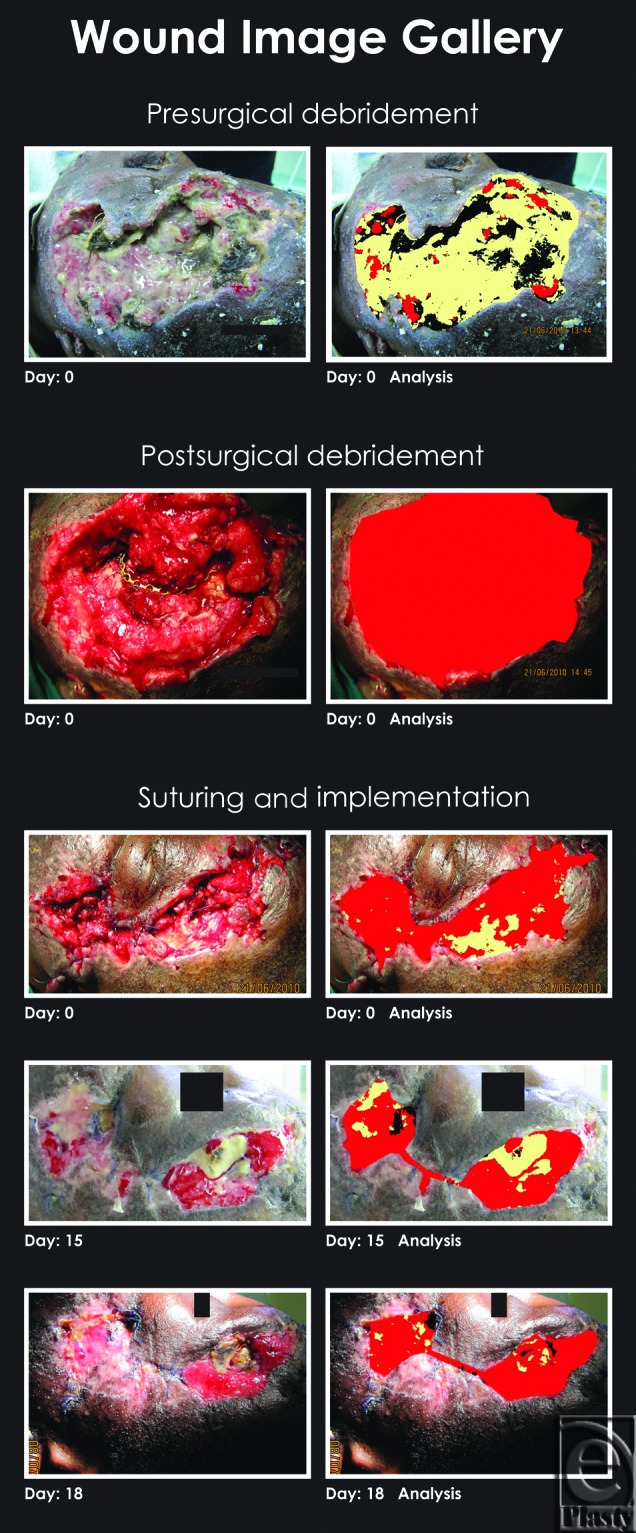
Wound image gallery tissue analysis: granulation tissue = red, slough = yellow, and necrotic tissue = black.

**Figure 2:**
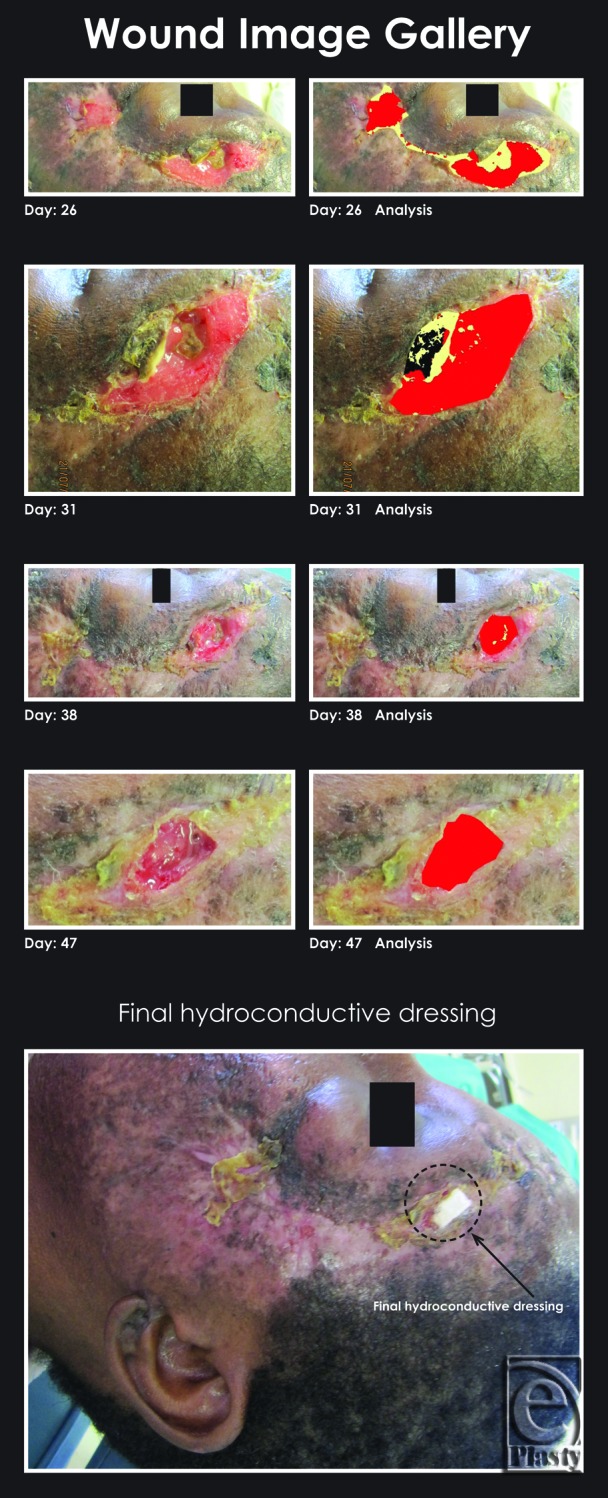
Wound image gallery tissue analysis continued with placement of the final piece of Drawtex dressing: granulation tissue = red, slough = yellow, and necrotic tissue = black.

Clinical examination revealed a fracture of the left lateral orbital wall, the floor of the orbit, and a comminuted fracture of the left zygoma. The patient presented with enophthalmos and trismus as well as an intraoral communication with the external degloving injury. There were no cranial nerve defects. Following an initial conservative debridement and reconstruction while attempting to preserve as much of the normal structure as possible, the wounds were dressed with a hydroconductive dressing. At the first dressing change in 24 hours, the wound appeared clean, so the decision was made to continue with the hydroconductive dressing change at 3-day intervals (Figures 1 and 2; days 0 to day 47). No topical or systemic antimicrobials were used. On discharge, the patient was able to function well with a reasonably good aesthetic result. She was subsequently lost to follow-up.

All photographs of the wound bed were taken at each dressing change by the same operator using the same digital camera with the same camera settings. The distance of the camera from the wound remained constant. Wound bed analysis from photographic images was done (Image care Ltd, London, United Kingdom) (Figures 1 and 2).

**Figure 3:**
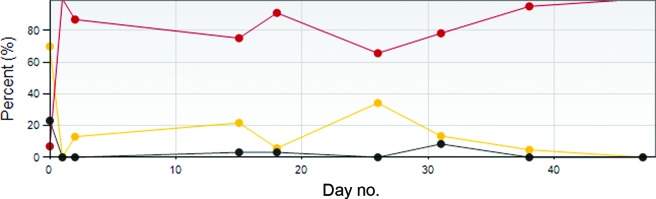
The tissue analysis graph depicts the percentage of granulation tissue = red, slough = yellow, and necrotic tissue = black.

Elixr, a statistical pattern-recognition algorithm that classifies each wound color pixel in a wound image, providing a documented area measurement variance of only 1% (with flat wound images) to 5% (with rounded wound images), was used. Accurate readings of granulation, slough, and eschar found in the wound bed were provided (Figure 3 and Table 1).

## RESULTS

Using only selective conservative debridement following bony reconstruction and repeated hydroconductive dressing changes, this severe injury healed with preservation of the important facial features. The wound showed signs of rapid healing by day 10 and near complete healing by day 52 with reepithelialization. On discharge, the patient was able to function well with a reasonably good aesthetic result. No further extensive surgical procedures were done.

## DISCUSSION

Debridement is an integral component for care of wounds of all etiologies that contain necrotic tissue, high bacterial burden, or other complicating unwanted elements.^5^ Surgical (sharp) debridement is the most rapid, direct, and effective method of debridement. However, not all wounds or patients are candidates for sharp debridement, especially if such debridement may remove or injure structures. Nonsurgical enzymatic debridement presents an alternative to sharp surgical debridement but is difficult to meticulously control. Also, data would indicate cautious approach when utilizing enzymatic agents in wounds with significant bacterial bioburdens, and they may predispose to worsening invasive infection, leading to sepsis.

In the case presented, the effectiveness of a hydroconductive dressing, Drawtex (SteadMed Medical, LLC, Fort Worth, Tex), with specialized LevaFiber technology in the management of avulsive facial injury has been demonstrated. The properties of the hydroconductive dressing are mainly its ability to remove bacteria and detrimental cytokines from the wound,^4^ its ability to extract nutrient-rich inflammatory exudates essential for biofilm survival,^6^ and the ability to apply a single layer or multiple layers of the hydroconductive dressing depending on the wound fluid burden.

**Figure 4:**
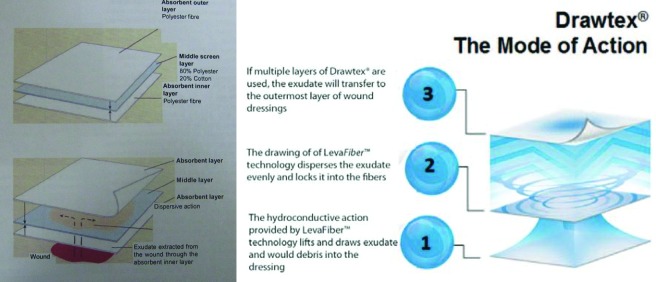
(left) Structure of Drawtex,^8^ and (right) mechanism of action of Drawtex illustrated.^7^

In addition to keeping the wound environment moist, drawing the exudate away from the wound surface removes toxic components, such as slough, necrotic tissue, and bacteria, that compromise wound healing. This reduces the risk of infection and maceration of the wound. By dispersing the exudate both horizontally and vertically, Drawtex controls and retains the wound fluid within its cross-action structure (Figure 4). The wound fluid is held in the dressing so it can be transferred to another layer of dressing if needed.

LevaFiber technology, the proprietary name of the Drawtex dressing technology, combines two types of absorbent, cross-action structures that facilitate the ability to move large volumes of fluid and other debris from the wound through the dressing (see the figure). This hydroconductive action allows the dressing to lift, hold, and transfer the wound exudate both horizontally and vertically into the dressing, where it can absorb 500% of its own weight. The hydroconductive action disperses the contents of the dressing and allows additional hydroconductive layers to be used for more heavily exuding wounds. The dressing also provides hydroconductive debridement that lifts and loosens adherent slough not absorbed into the dressing, allowing it to migrate toward the dressing for easy removal when the dressing is changed.

These characteristics make this hydroconductive dressing a versatile alternate to traditional wound management protocols by creating an ideal environment for healing to take place.

## IT SHOULD NOT BE USED IN ARTERIAL BLEEDING

It can be used in conjunction with gels and ointments and maintains its integrity when saturated. It is superabsorbent and works against gravity.

This case report demonstrates further that a hydroconductive dressing can be useful for traumatic avulsive injuries and can remove deterrents to wound healing without resorting to therapies that might result in additional loss of tissue in situations where maximal tissue preservation is paramount.

## Figures and Tables

**Table 1:** Wound measurement results

Day no.	*a*	*p*	*w x h*	Status/whr, %	Granulation tissue, %	Slough, %	Necrotic tissue, %	Date	No. of slides studied
47	…	…	…	…	100.0	0.0	0.0	August 6, 2010	9
38	…	…	…	…	96.0	4.0	0.0	July 28, 2010	8
31	…	…	…	…	78.3	13.4	8.4	July 21, 2010	7
26	…	…	…	…	65.7	34.3	0.0	July 16, 2010	6
18	…	…	…	…	91.2	5.7	3.1	July 8, 2010	5
15	…	…	…	…	75.2	21.7	3.1	July 5, 2010	4
0	…	…	…	…	87.0	13.0	0.0	June 21, 2010	3
0	…	…	…	…	100.0	0.0	0.0	June 21, 2010	2
0	…	…	…	…	6.9	70.0	23.1	June 21, 2010	1
